# Associations between Serum Sex Hormone Concentrations and Whole Blood Gene Expression Profiles in the General Population

**DOI:** 10.1371/journal.pone.0127466

**Published:** 2015-05-22

**Authors:** Robin Haring, Claudia Schurmann, Georg Homuth, Leif Steil, Uwe Völker, Henry Völzke, Brian G. Keevil, Matthias Nauck, Henri Wallaschofski

**Affiliations:** 1 Institute for Clinical Chemistry and Laboratory Medicine, University Medicine Greifswald, Greifswald, Germany; 2 DZHK (German Centre for Cardiovascular Research), partner site Greifswald, Greifswald, Germany; 3 Interfaculty Institute for Genetics and Functional Genomics, University Medicine Greifswald, Greifswald, Germany; 4 Institute for Community Medicine; University Medicine Greifswald, Greifswald, Germany; 5 Department of Clinical Chemistry, University Hospital South Manchester, Manchester, United Kingdom; Northern Institute for Cancer Research, UNITED KINGDOM

## Abstract

**Background:**

Despite observational evidence from epidemiological and clinical studies associating sex hormones with various cardiometabolic risk factors or diseases, pathophysiological explanations are sparse to date. To reveal putative functional insights, we analyzed associations between sex hormone levels and whole blood gene expression profiles.

**Methods:**

We used data of 991 individuals from the population-based Study of Health in Pomerania (SHIP-TREND) with whole blood gene expression levels determined by array-based transcriptional profiling and serum concentrations of total testosterone (TT), sex hormone-binding globulin (SHBG), free testosterone (free T), dehydroepiandrosterone sulfate (DHEAS), androstenedione (AD), estradiol (E2), and estrone (E1) measured by liquid chromatography-tandem mass spectrometry (LC-MS/MS) and immunoassay. Associations between sex hormone concentrations and gene expression profiles were analyzed using sex-specific regression models adjusted for age, body mass index, and technical covariables.

**Results:**

In men, positive correlations were detected between AD and *DDIT4 *mRNA levels, as well as between SHBG and the mRNA levels of *RPIA*, *RIOK3*, *GYPB*, *BPGM*, and *RAB2B*. No additional significant associations were observed.

**Conclusions:**

Besides the associations between AD and *DDIT4* expression and SHBG and the transcript levels of *RPIA*, *RIOK3*, *GYPB*, *BPGM*, and *RAB2B*, the present study did not indicate any association between sex hormone concentrations and whole blood gene expression profiles in men and women from the general population.

## Introduction

Total testosterone (TT), dehydroepiandrosterone sulfate (DHEAS), androstenedione (AD), estradiol (E2), and estrone (E1) are major sex hormones of the human systemic circulation, regulating a wide range of metabolic and hemodynamic effects [1]. Biologically, TT is the most important sex hormone in men essentially needed for the development and maintenance of specific reproductive tissues, such as the testis, and responsible for megakaryocytes and platelet development and production [2], as well as erythropoiesis [3,4]. The primary female sex hormones E1 and E2 are mainly produced by the ovaries. They regulate reproductive functions, bone formation [5], immune function [6], hemostasis, and erythropoiesis [7]. Various epidemiological studies reported a multitude of associations between the concentrations of sex hormones as well as sex hormone-binding protein (SHBG, the key protein for binding and transporting of sex hormones) and cardiometabolic risk factors including obesity, metabolic syndrome, type 2 diabetes mellitus [8], and mortality [9]. However, pathophysiological explanations and insights into possible regulatory malfunction are sparse and limited to cell culture or animal models [10]. Thus, we used an established workflow [11] to analyze 991 men and women from the general population for significant correlations between the concentrations of different serum sex hormones on the one hand and whole blood mRNA levels determined by array-based transcriptional profiling on the other hand.

## Methods

### Study Population

The Study of Health in Pomerania (SHIP-TREND) is a population-based cohort study in West Pomerania, a region in the northeast of Germany. Study design and sampling methods were described previously [12]. Of a stratified random net sample of 8,826 individuals aged 20–79 years with German citizenship and main residency in the study area, 4,420 individuals (2,145 men) finally participated (response 50.1%) in baseline examinations from September 2008 to September 2012. Measurement of gene expression profiles was limited to the first 1.000 suitable SHIP-TREND participants (555 women) that fasted for at least 10 hours prior to blood donation, with slight variations in sample size dependent on sex hormone data availability. All participants gave informed written consent. The study protocol is consistent with the principles of the Declaration of Helsinki, as reflected by an a priori approval of the Ethics Committee of the University of Greifswald.

### Anthropometric and sex hormone measurements

Body mass index (BMI) was calculated as weight in kilograms divided by height in square meters measured according to standard protocols using digital scales (Seca 862, Seca Germany) and a measuring stick (Seca 220, Seca, Germany). After a minimum fasting period of at least 10 hours, fasting blood samples were taken from the cubital vein in the supine position and prepared for immediate analysis or for storage at -80°C for further analysis. Serum concentrations of TT, AD, E2, and E1 were measured from frozen aliquots using liquid chromatography-tandem mass spectrometry (LC-MS/MS), as previously described in detail [13,14]. In brief, stable isotopes of analytes were used as internal standards. Intra- and inter-assay coefficients of variation were < 10% over the measurement range of 0.3–35 nmol/l. The lower limit of quantitation was 0.25 nmol/l. For E2, the inter-assay imprecision were 5.4, 3.7 and 4.9% and for E1 5.3, 3.8 and 5.1% at concentrations of 125, 400 and 1500 pmol/l, respectively. The intra-assay imprecision for these concentrations were 3.1, 3.5 and 4.0% for E2 and 4.0, 3.4 and 5.0% for E1, respectively. All means were within 8% of the PBS-based quality control targets. The measurement range for E2 and E1 was 25–2000 pmol/l. The lower limit of detection was 8.0 and 3.9 pmol/l for E2 and E1, respectively. The lower limits of quantitation were 10 pmol/l for E2 and 6 pmol/l for E1. Concentrations of SHBG and DHEAS were measured using competitive chemiluminescent immunoassays on an Immulite 2000 XPi (Siemens Healthcare Diagnostics, Eschborn, Germany) with an inter-assay coefficient of variation of 3.5% and 8.3% in the low pool, and 4.8% and 5.4% in the high pool, respectively. Free T was calculated based on measured TT and SHBG concentrations and a standard albumin concentration based on the formula published by Vermeulen et al. [15].

### Gene expression data

RNA was prepared from whole blood collected and stored in PAXgene Blood RNA Tubes (BD, Heidelberg, Germany) using the PAXgene Blood miRNA Kit (Qiagen, Hilden, Germany). Isolation of RNA was performed using a QIAcube according to protocols provided by the manufacturer Qiagen. Purity and concentration of RNA were determined using a NanoDrop ND-1000 UV-Vis Spectrophotometer (Thermo Scientific, Hennigsdorf, Germany). To ensure a consistently high RNA quality, all preparations were analyzed using RNA 6000 Nano LabChips on a 2100 Bioanalyzer (both from Agilent Technologies, Santa Clara, CA, USA) according to the manufacturer's instructions. Samples exhibiting a RNA integrity number (RIN) less than seven were excluded from subsequent analyses. The Illumina TotalPrep-96 RNA Amplification Kit (Ambion, Darmstadt, Germany) was used for reverse transcription of 500 ng RNA into double-stranded (ds) cDNA and subsequent synthesis of biotin-UTP-labeled antisense-cRNA using this cDNA as the template. Finally, in total 3,000 ng of cRNA were hybridized with a single array on the Illumina HumanHT-12 v3 BeadChips, followed by washing and detection steps in accordance with the Illumina protocol. BeadChips were scanned using the Illumina Bead Array Reader. Further details on expression data transformation and quality control are available elsewhere [11].

### Statistical analysis

To reduce heteroscedasticity, all sex hormone variables were transformed using the natural logarithm. To investigate correlations between sex hormone concentrations and mRNA levels, we performed linear regression analyses, separately for men and women. Here, gene expression levels were regressed on the respective sex hormone concentrations with adjustment for age, BMI, and technical covariables including RNA quality (RIN), plate layout after RNA amplification, and sample storage time (time between blood donation and RNA preparation) [11]. To test the robustness of our findings, linear regression models were additionally adjusted for blood cell counts (white and red blood cell count, lymphocyte, neutrophil, monocyte, eosinophil, and basophil counts, as well as hematocrit, and platelet counts). To correct for multiple testing the Benjamini and Hochberg false discovery rate (FDR) method was used. Associations with a FDR < 1% were considered statistically significant. All statistical analyses were performed using the computing environment R (http://www.R-project.org).

To identify functional connections between the implicated genes and test for a possible overrepresentation of certain gene sets, we used Ingenuity Pathway Analysis (IPA) software (Ingenuity Systems, build version: 220217, content version: 16542223, release date 2013-05-13, www.ingenuity.com), based on all transcripts associated with the respective phenotype (p-value <1x10^-3^). IPA accesses a comprehensive database containing various genes and gene products that interact with each other to select pathways and functions enriched by the input genes. It was used to test the set of significantly associated genes for enrichment in defined canonical pathways and categories of biological functions, respectively. For genes with more than one probe, the respective probe with the lowest p-value was used. After mapping the Illumina probes within IPA to gene names, we were not able to detect any significant enrichment in any canonical pathway or function for the analyzed phenotypes.

Finally, we performed a lookup for expression quantitative trait loci (eQTL) in the “blood eQTL browser” (http://genenetwork.nl/bloodeqtlbrowser/) of all previously published Genome-wide association studies (GWAS) SNPs (http://www.genome.gov/gwastudies/) for the sex hormone traits of interest in our study [16].

## Results

Baseline characteristics of the study population were presented by sex (Table 1). Linear regression models adjusted for age, BMI, and technical covariables did not reveal any genes whose mRNA levels significantly (FDR < 1%) correlated with sex hormone concentrations including TT, free T, SHBG, DHEAS, E1, and E2 in both men and women (Table 2). Analyses of AD in men yielded a positive correlation with *DDIT4* mRNA levels. Additional adjustment for blood cell counts altered none of the findings, except for SHBG concentrations in men. After adjustment for age, BMI, technical covariables, and blood cell count, five significant, previously undetected hits were identified: *RPIA*, *RIOK3*, *GYPB*, *BPGM*, and *RAB2B*. Additional sensitivity analyses were performed after 1) stratifying the female study sample by menopausal status (pre- vs. post-menopausal), 2) the exclusion of 112 women and one man using exogenous hormones, and 3) after the exclusion of 31 women using hormone replacement therapy in particular, but without any substantial change of the revealed estimates (all results with a FDR < 0.1 are provided in S1 Table).

**Table 1:** Baseline characteristics of the study population by sex.

	Men [N = 436]	Women [N = 555]	P-value *
Age, years	50.0 (39.0, 61.0)	51.0 (40.5, 60.0)	0.880
Body mass index, kg/m^2^	27.7 (25.0, 30.2)	26.3 (23.1, 30.0)	<0.001
Menopausal status (post-menopausal), %	N.A.	36.8	
Sex hormone concentrations			
Total testosterone, nmol/l	17.30 (14.23, 20.53)	0.80 (0.59, 1.03)	<0.001
Sex hormone-bindi ng globulin, nmol/l	38.4 (28.6, 45.8)	64.1 (42.8, 79.7)	<0.001
Free testosterone, nmol/l	0.456 (0.280, 0.400)	0.011 (0.007, 0.013)	<0.001
Dehydroepiandrosterone sulfate, mg/l	1.86 (1.01, 2.49)	1.16 (0.64, 1.50)	<0.001
Androstenedione, nmol/l	2.77 (2.17, 3.71)	2.27 (1.64, 3.36)	<0.001
Estradiol, pmol/l	76.5 (60.6, 91.4)	203.4 (63.3, 412.9)	<0.001
Estrone, pmol/L	115.5 (94.9, 148.3)	110.7 (71.6, 214.7)	0.703
Blood cell counts, N			
White blood cell	5.3 (4.6, 6.4)	5.5 (4.8, 6.5)	0.139
Red blood cell	4.9 (4.6, 5.1)	4.4 (4.2, 4.7)	<0.001
Lymphocyte	30.0 (25.4, 34.9)	30.0 (25.0, 35.1)	0.859
Neutrophil	56.4 (51.6, 61.9)	58.8 (53.3, 64.4)	<0.001
Monocyte	9.6 (8.2, 11.2)	8.1 (7.0, 9.6)	<0.001
Eosinophil	2.3 (1.6, 3.6)	2.0 (1.3, 3.0)	<0.001
Basophil	0.4 (0.2, 0.6)	0.4 (0.3, 0.6)	0.715
Hematocrit	0.4 (0.4, 0.5)	0.4 (0.4, 0.4)	<0.001
Platelet	208 (182, 237)	233 (202, 271)	<0.001

The presented data shows the median and the corresponding interquartile range.

Differences between men and women were tested using a two-sided Mann-Whitney U test.

The absolute number of 991 participants with complete gene expression data varied due to missing sex hormone data:

N = 985 for total testosterone and androstenedione, N = 946 for SHBG and free T, N = 971 for DHEAS, N = 665 for estradiol, and N = 966 for estrone.

Values for measured sex hormones concentrations and blood cell counts are reported and cell type measurement, respectively.

**Table 2:** Gene expression analysis of sex hormone concentrations in men and women.

	Men		Women	
	# probes FDR < 0.01	Gene name	# probes FDR < 0.01	Gene name
Total testosterone	0		0	
Sex hormone-binding globulin	0		0	
Free testosterone	0		0	
Dehydroepiandrosterone sulfate	0		0	
Androstenedione	1	*DDIT4*	0	
Estradiol	0		0	
Estrone	0		0	

Regression models were adjusted for age, BMI, and technical covariables including RNA quality (RIN), plate layout after RNA amplification, and sample storage time.

To account for multiple testing the Benjamini and Hochberg false discovery rate (FDR) method was used.

The lookup for possible *cis*- and *trans*-eQTLs in the “blood eQTL browser” revealed that none of the gene-specific transcripts detected to be associated with serum sex hormone concentrations in our analysis, namely of *DDIT4*, *RPIA*, *RIOK3*, *GYPB*, *BPGM*, and *RAB2B*, represents a *cis*- or *trans*-eQTL of the lead SNPs identified in the respective GWAS, at least not in whole blood (S2 Table).

## Discussion

The present analysis on correlations between whole blood gene expression and serum sex hormone concentrations yielded little sex hormone-associated differences in mRNA levels among men and women from the general population. In men, AD, a precursor of more potent sex hormones including TT, E1, and E2, was correlated with the expression of *DDIT4* (DNA-damage-inducible transcript 4), encoding an inhibitor of mTOR signaling also known as *REDD1*. With respect to SHBG concentrations in men, *RPIA*, *RIOK3*, *GYPB*, *BPGM*, and *RAB2B* exhibited significantly correlated transcript levels after adjustment for age, BMI, technical covariables, and blood cell counts. These genes encode ribose 5-phosphate isomerase A, RIO kinase 3, glycophorin B, 2,3-bisphosphoglycerate mutase, and the member of the RAS oncogene family RAB2B, respectively. To the best of our knowledge, for none of the mentioned proteins a plausible link to the serum concentrations of the corresponding sex hormones can be derived from the available literature. However, in case of the positively SHBG-correlated *RPIA*-, *RIOK3*-, *GYPB*-, *BPGM*-, and *RAB2B*-specific transcripts, it is noteworthy that all these gene are particularly strong expressed in CD71-positive early erythroid cells according to the BioGPS expression browser (http://www.biogps.org). As this observation might be transferable to reticulocytes, the significant positive correlation between SHBG serum concentration and the mRNA levels of these five genes could indicate a higher reticulocyte percentage among the total red blood cells to be correlated with serum SHBG. However, as reticulocyte counts are not available in SHIP, this postulated relationship remains speculative.

As genetic variants associated with serum hormone concentrations could exert their effects by modulating the expression strength of other genes in *cis* or *trans*, the detection of such corresponding eQTL relationships in our analysis was conceivable. However, of the six genes detected in our study to exhibit mRNA levels significantly correlated with serum sex hormone concentrations, namely with AD and SHBG, neither represented a described associated eQTL of the lead SNPs of the corresponding GWAS. The other way around, none of the genes described to represent eQTLs significantly associated with the lead SNPs of the GWAS on serum sex hormone concentrations (S2 Table) turned out to exhibit mRNA levels correlated with sex hormone concentrations in our study. The fact that the latter eQTLs were not detected in our study could be due to different reasons: First, the statistical power might be too low due to our limited sample size. Second, these eQTLs have been identified in whole blood and may therefore at least partially not be functionally involved in the production or degradation of sex hormones in the relevant tissues. And third, generally not all detected eQTLs might be of real physiological relevance.

Given the small effect sizes of the identified common variants associated with serum sex hormone concentrations in recent GWAS [17–20], genetic variants appear to explain only a modest fraction of the inter-individual variation in sex hormone concentrations. Therefore, gene expression studies could be predicted to represent a different promising approach for the identification of biological pathways linked to sex hormone regulation and the analysis of associated gene regulatory networks. But despite the indisputable importance of sex hormones as biologically relevant, informative, and constituent parts of human physiology, the widely negative findings of the present study substantiate the discussion about the limitations of gene expression analysis restricted to whole blood cell samples. Even though the easily accessible whole blood cells are known to express a representative proportion (> 80%) of the human genome [21], and can therefore be used to detect biomarkers of several human traits and diseases [22], gene expression patterns measured in whole blood might not directly translate to other tissues more closely related to the trait of interest such as total testosterone or other sex hormones. On a similar note, another previous analysis of a random sample of 285 post-menopausal women, based on non-fasting peripheral blood samples, also detected no profound gene expression signatures for concentrations of TT, SHBG, E2, FSH, and progesterone [23]. Taken together, the failure of detection of correlations between gene expression and sex hormones in the present study may reflect the comparably healthy subsample of our population-based cohort study, including a low variability in sex hormone concentrations, as well as the inherent limitations of gene expression analysis based on peripheral blood. Thus, repeated expression analyses in specific patient populations based on specific tissues warrant further research.

## Supporting Information

S1 Table Regression estimates of gene expression analysis of sex hormone concentrations in men and women with Benjamini-Hochberg FDR < 10%. (DOCX)

S2 Table Expression quantitative trait loci (eQTL) look-up of previously published GWAS SNPs. (DOCX)
