# Supplementary material for: Associations between Serum Sex Hormone Concentrations and Whole Blood Gene Expression Profiles in the General Population
Source: PLoS One. 2015 May 22;10(5):e0127466. doi: 10.1371/journal.pone.0127466 (PMC4441431; doi:10.1371/journal.pone.0127466)
Supplement: S1 Table — (DOCX) [file pone.0127466.s001.docx]

Supplemental Table 1: Regression estimates of gene expression analysis of sex hormone concentrations in men and women with Benjamini-Hochberg FDR < 10%.


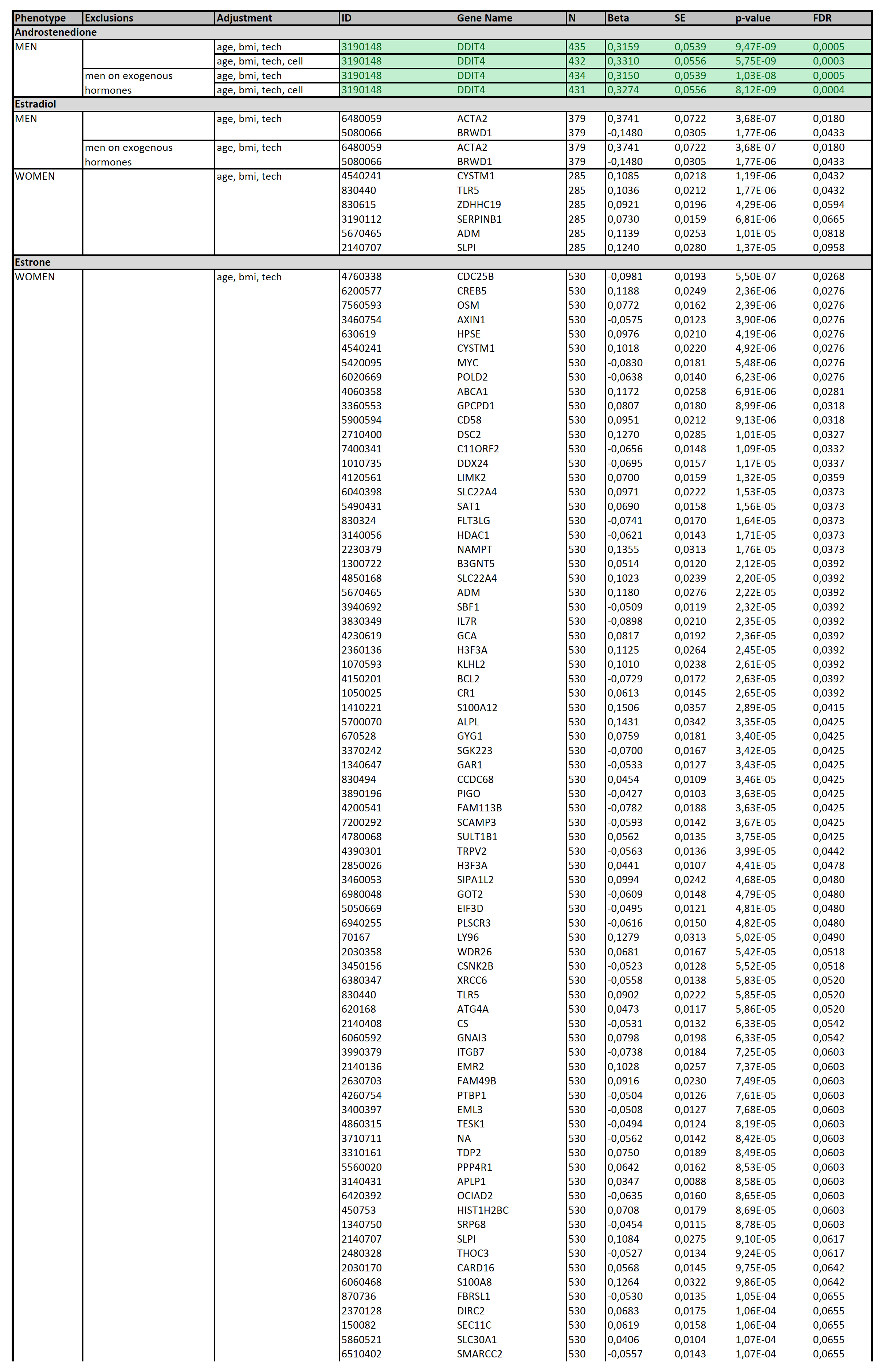


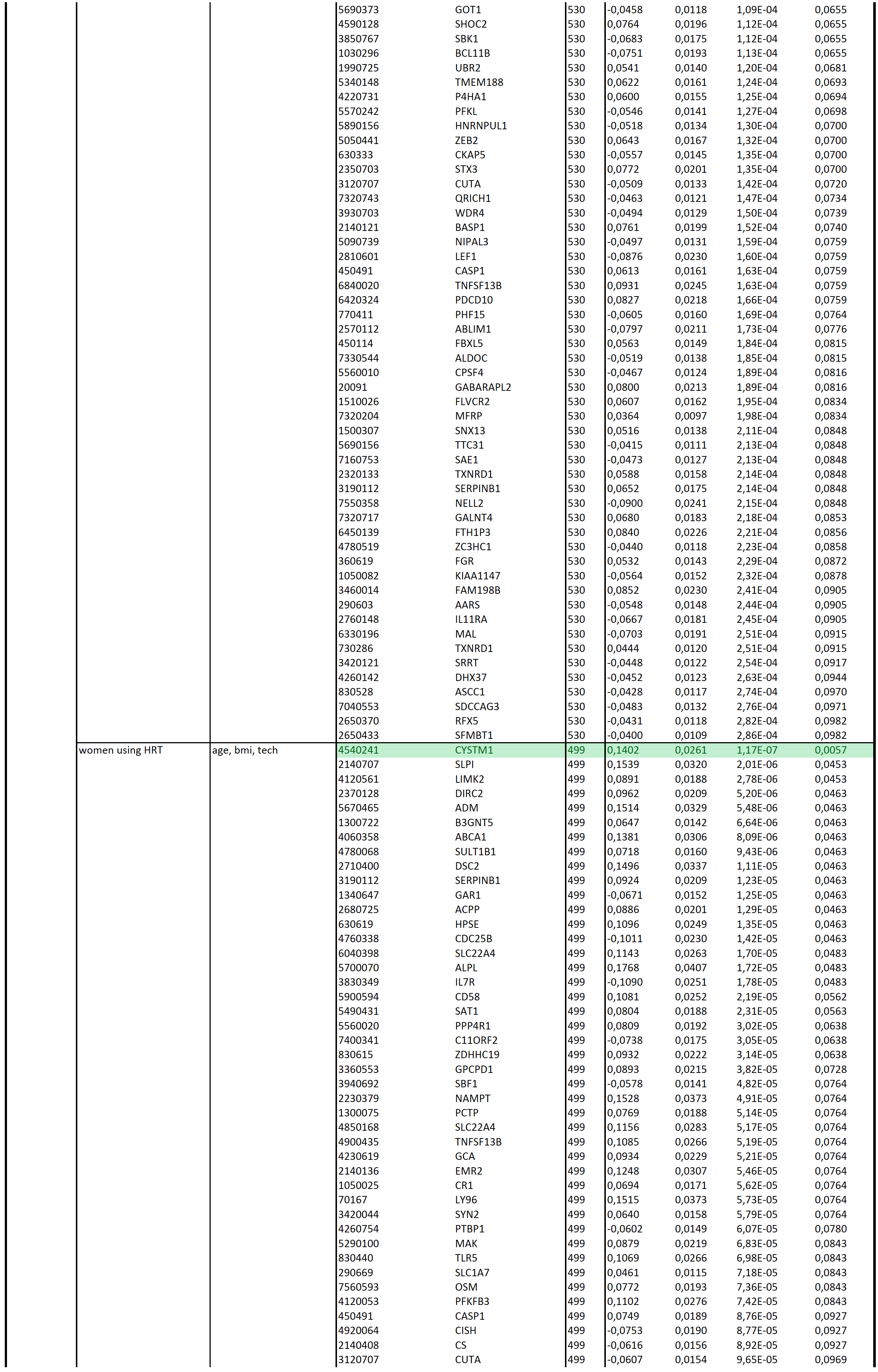


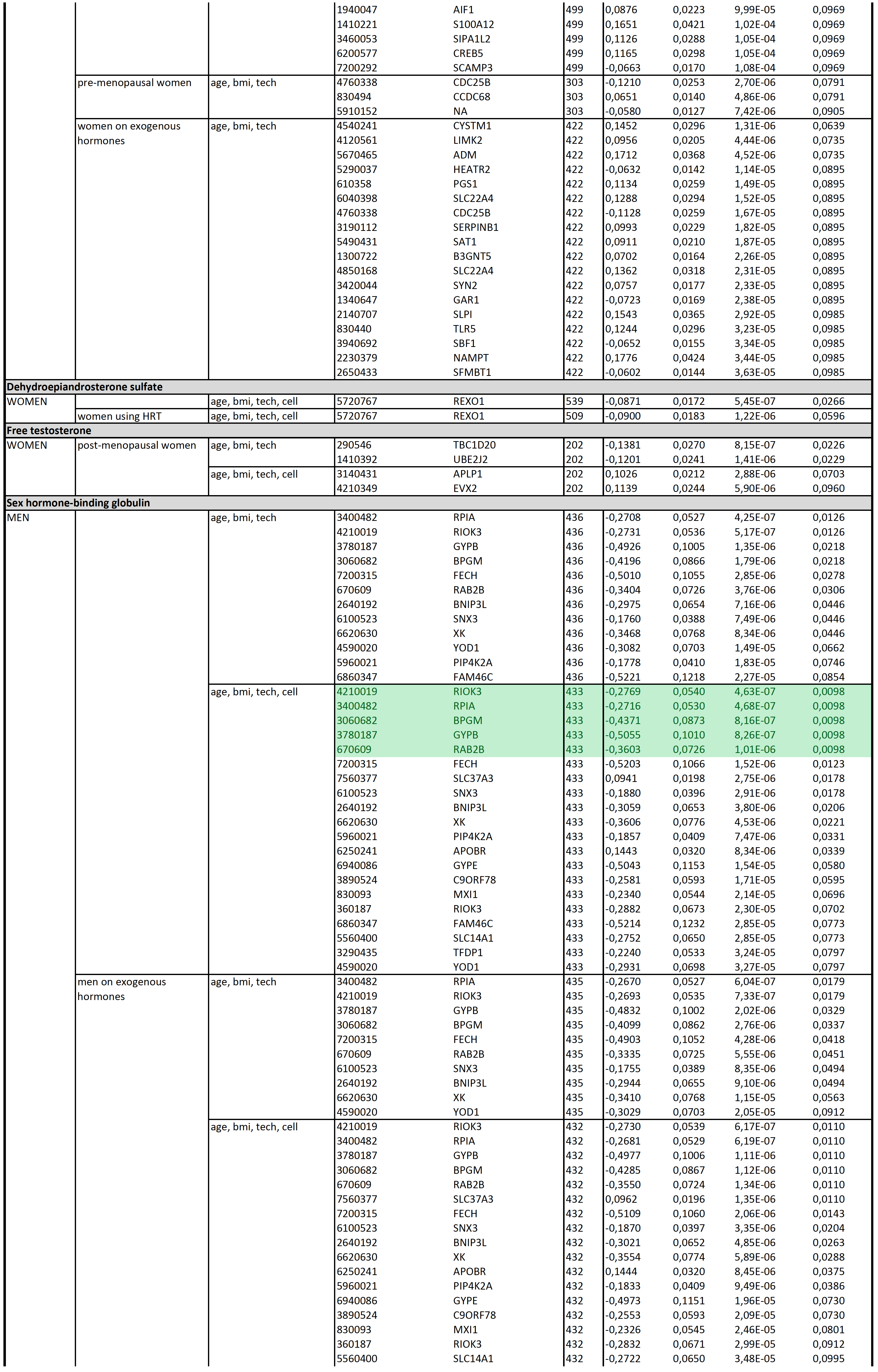


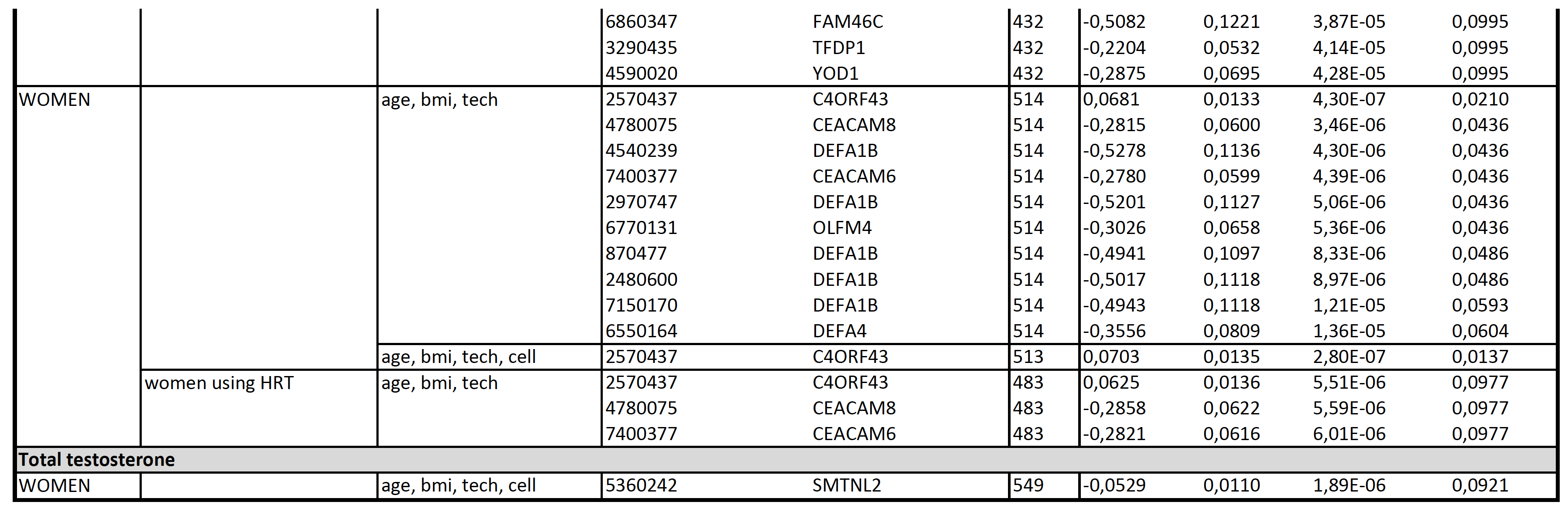


Regression models were adjusted for age, body mass index (bmi), and technical covariables (tech) including RNA quality, plate layout after RNA amplification, and sample storage time, and blood cell counts (cell).

To correct for multiple testing the Benjamini and Hochberg false discovery rate (FDR) method was used.

ID, Illumina Array Address ID; SE, standard error.
