# Supplementary material for: Associations between Serum Sex Hormone Concentrations and Whole Blood Gene Expression Profiles in the General Population
Source: PLoS One. 2015 May 22;10(5):e0127466. doi: 10.1371/journal.pone.0127466 (PMC4441431; doi:10.1371/journal.pone.0127466)
Supplement: S2 Table — (DOCX) [file pone.0127466.s002.docx]

**Supplemental Table 2.** Expression quantitative trait loci (eQTL) look-up of previously published GWAS SNPs.

| **Known GWAS hits** | | | | | | **eQTL lookup** | | | | | | | |
| --- | --- | --- | --- | --- | --- | --- | --- | --- | --- | --- | --- | --- | --- |
| **Strongest SNP & Risk Allele** | **OR / Beta** | **p-value** | **Chr-ID** | **Chr_pos** | **Reported Gene(s)** | **cis/trans** | **Gene Name** | **Probe Chr** | **ProbeCenter ChrPos** | **p-value** | **FDR** | **Z-score** | **Assessed Allele** |
| **Dehydroepiandrosterone sulphate (PMID: 21533175)** | | | | | | | | | | | | | |
| rs6738028-G | 0,04 | 2,0E-08 | 2 | 111.191.750 | BCL2L11 | - |  |  |  |  |  |  |  |
| rs740160-T | 0,15 | 2,0E-16 | 7 | 99.360.257 | ARPC1A | - |  |  |  |  |  |  |  |
| rs11761528-T | 0,16 | 3,0E-36 | 7 | 99.521.178 | ZKSCAN5 | *cis* | CPSF4 | 7 | 98.892.860 | 2,4E-07 | 9,41915E-05 | 5,17 | T |
| rs17277546-A | 0,11 | 2,0E-11 | 7 | 99.891.948 | TRIM4, CYP3A43 | *cis* | TRIM4 | 7 | 99.326.657 | 5,0E-21 | 0 | -9,41 | A |
| rs2497306-C | 0,04 | 5,0E-09 | 10 | 92.725.454 | HHEX | *trans* | CD160 | 1 | 144.407.391 | 6,9E-13 | 0 | -7,18 | A |
|  |  |  |  |  |  | *trans* | FEZ1 | 11 | 124.821.046 | 1,6E-10 | 0,000306279 | -6,39 | A |
|  |  |  |  |  |  | *cis* | HHEX | 10 | 94.445.158 | 2,9E-15 | 0 | 7,89 | A |
| rs2185570-C | 0,06 | 2,0E-08 | 10 | 94.991.513 | CYP2C9 | *cis* | PDLIM1 | 10 | 96.987.526 | 1,1E-07 | 3,20359E-05 | 5,31 | C |
| rs7181230-G | 0,05 | 5,0E-11 | 15 | 40.068.540 | BMF | *-* |  |  |  |  |  |  |  |
| rs2637125-A | 0,09 | 3,0E-19 | 19 | 47.898.636 | SULT2A1 | *cis* | CABP5 | 19 | 53.225.187 | 3,4E-06 | 0,001405797 | -4,65 | A |
| **Estradiol (breast cancer) (PMID: 23518928)** | | | | | | | | | | | | | |
| rs1864729 | 0,13 | 3,0E-08 | 8 | 97.269.961 | TSPYL5 | - |  |  |  |  |  |  |  |
| **Sex hormone-binding globulin (PMID: 22829776)** | | | | | | | | | | | | | |
| rs17496332-A | 0,03 | 1,0E-11 | 1 | 107.003.753 | PRMT6 | *cis* | PRMT6 | 1 | 107.402.980 | 8,4E-139 | 0 | -25,08 | G |
|  |  |  |  |  |  | *cis* | PRMT6 | 1 | 107.402.830 | 9,1E-55 | 0 | -15,59 | G |
| rs780093-T | 0,03 | 2,0E-16 | 2 | 27.519.736 | GCKR | *cis* | SNX17 | 2 | 27.453.289 | 1,0E-10 | 0 | 6,47 | T |
|  |  |  |  |  |  | *cis* | NRBP1 | 2 | 27.518.384 | 2,7E-05 | 0,012290503 | 4,20 | T |
| rs293428-A | 0,03 | 3,0E-08 | 4 | 68.726.064 | UGT2B15 | *-* |  |  |  |  |  |  |  |
| rs3779195-A | 0,03 | 3,0E-08 | 7 | 98.364.050 | BAIAP2L1 | *-* |  |  |  |  |  |  |  |
| rs440837-A | 0,03 | 3,0E-09 | 8 | 80.549.739 | ZBTB10 | *-* |  |  |  |  |  |  |  |
| rs7910927-T | 0,05 | 6,0E-35 | 10 | 63.379.150 | JMJD1C | *cis* | - | 10 | 64.591.825 | 2,9E-05 | 0,013124751 | 4,18 | T |
| rs4149056-T | 0,03 | 2,0E-08 | 12 | 21.178.615 | SLCO1B1 | *-* |  |  |  |  |  |  |  |
| rs8023580-T | 0,03 | 8,0E-12 | 15 | 96.165.062 | NR2F2 | *-* |  |  |  |  |  |  |  |
| rs12150660-T | 0,1 | 2,0E-106 | 17 | 7.618.597 | SHBG | *cis* | TNFSF12 | 17 | 7.401.715 | 1,5E-149 | 0 | 26,05 | T |
|  |  |  |  |  |  | *cis* | EIF4A1,CD68 | 17 | 7.423.902 | 1,7E-106 | 0 | -21,92 | T |
|  |  |  |  |  |  | *cis* | SAT2 | 17 | 7.470.505 | 2,8E-18 | 0 | -8,72 | T |
|  |  |  |  |  |  | *cis* | TNFSF12,EIF4A1 | 17 | 7.404.948 | 1,3E-06 | 0,000389747 | 4,85 | T |
| rs6258-T | 0,27 | 3,0E-46 | 17 | 7.631.360 | SHBG | *-* |  |  |  |  |  |  |  |
| rs1641537-T | 0,06 | 1,0E-24 | 17 | 7.642.403 | SHBG | *cis* | EIF4A1,CD68 | 17 | 7.423.902 | 3,2E-20 | 0 | 9,21 | T |
|  |  |  |  |  |  | *cis* | TNFSF12 | 17 | 7.401.715 | 1,9E-11 | 0 | -6,71 | T |
| rs1625895-T | 0,12 | 2,0E-21 | 17 | 7.674.797 | SHBG | *-* |  |  |  |  |  |  |  |
| rs2411984-A | 0,03 | 4,0E-14 | 17 | 49.368.389 | ZNF652 | *cis* | PHOSPHO1 | 17 | 44.656.026 | 4,7E-18 | 0 | -8,66 | A |
|  |  |  |  |  |  | *cis* | GNGT2 | 17 | 44.638.916 | 8,0E-13 | 0 | -7,16 | A |
| rs1573036-T | 0,03 | 4,0E-14 | 23 | 110.576.840 | TDGF3 | *-* |  |  |  |  |  |  |  |
| **Total testosterone (PMID: 21998597)** | | | | | | | | | | | | | |
| rs12150660-T | 31,8 | 1,0E-41 | 17 | 7.618.597 | SHBG | *cis* | TNFSF12 | 17 | 7.401.715 | 1,5E-149 | 0 | 26,05 | T |
|  |  |  |  |  |  | *cis* | EIF4A1,CD68 | 17 | 7.423.902 | 1,7E-106 | 0 | -21,92 | T |
|  |  |  |  |  |  | *cis* | SAT2 | 17 | 7.470.505 | 2,8E-18 | 0 | -8,72 | T |
|  |  |  |  |  |  | *cis* | TNFSF12,EIF4A1 | 17 | 7.404.948 | 1,3E-06 | 0,000389747 | 4,85 | T |
| rs6258-T | 82,3 | 2,0E-22 | 17 | 7.631.360 | SHBG | *-* |  |  |  |  |  |  |  |

To account for multiple testing the Benjamini and Hochberg false discovery rate (FDR) method was used.

GWAS, genome-wide association study; eQTL, expression quantitative trait loci; Chr, chromosome; PMID, PubMed ID.
